# Allosteric conformational change cascade in cytoplasmic dynein revealed by structure-based molecular simulations

**DOI:** 10.1371/journal.pcbi.1005748

**Published:** 2017-09-11

**Authors:** Shintaroh Kubo, Wenfei Li, Shoji Takada

**Affiliations:** 1 Department of Biophysics, Graduate School of Science, Kyoto University, Kyoto, Japan; 2 National Laboratory of Solid State Microstructure and Department of Physics, Nanjing University, Nanjing, China; Weill Medical College of Cornell University, UNITED STATES

## Abstract

Cytoplasmic dynein is a giant ATP-driven molecular motor that proceeds to the minus end of the microtubule (MT). Dynein hydrolyzes ATP in a ring-like structure, containing 6 AAA+ (ATPases associated with diverse cellular activities) modules, which is ~15 nm away from the MT binding domain (MTBD). This architecture implies that long-distance allosteric couplings exist between the AAA+ ring and the MTBD in order for dynein to move on the MT, although little is known about the mechanisms involved. Here, we have performed comprehensive molecular simulations of the dynein motor domain based on pre- and post- power-stroke structural information and in doing so we address the allosteric conformational changes that occur during the power-stroke and recovery-stroke processes. In the power-stroke process, the N-terminal linker movement was the prerequisite to the nucleotide-dependent AAA1 transition, from which a transition cascade propagated, on average, in a circular manner on the AAA+ ring until it reached the AAA6/C-terminal module. The recovery-stroke process was initiated by the transition of the AAA6/C-terminal, from which the transition cascade split into the two directions of the AAA+ ring, occurring both clockwise and anti-clockwise. In both processes, the MTBD conformational change was regulated by the AAA4 module and the AAA5/Strut module.

## Author summary

The linear molecular motor dynein is an intriguing allosteric model protein. ATP hydrolysis, catalyzed by modules in the AAA+ ring, regulates the binding to the rail molecule, microtubule, which is ~15 nm away from the AAA+ ring. The molecular mechanisms underpinning this long-distance communication are unclear. Based on recently solved pre- and post- power-stroke crystal structure information, we performed, for the first time to our knowledge, molecular simulations of complete conformational changes between the two structures. The simulation revealed that module-by-module allosteric conformational changes occur. Interestingly, the transition cascade from the pre- to the post-power-stroke states propagated in a circular manner around the AAA+ ring, while that of the recovery transitions propagated in a bi-directional manner around the ring.

## Introduction

Dynein is an ATP-hydrolysis driven molecular motor that linearly proceeds along the microtubule (MT) towards its minus end [1–3]. Cytoplasmic dynein is involved in the transport of many different types of cargo such as mRNAs, proteins, and vesicles [4,5] and is also involved in mitosis [6,7]. Of the two forms, cytoplasmic dynein-1 is the major form, and is the focus of this work. Unless otherwise denoted, we simply denote cytoplasmic dynein-1 as “dynein”. Mutations in dynein are associated with various diseases such as amyotrophic lateral sclerosis (ALS) and cancer [8,9].

The motility of dynein is often compared with that of kinesin, a better-understood major MT-based molecular motor. While most kinesin moves towards the plus end of the MT, dynein moves towards its minus end. Most kinesin molecules do not move backwards, but it is known that yeast dynein, for example, moves stochastically with about 20% molecules moving backward [10–12]. The step size is always 8 nm, the structural unit of MT [13], for kinesin but varies in the range of 8–32 nm for dynein [10–12,14]. The homo-dimeric kinesin is known to move in a hand-over-hand manner [15]: Starting from the two-head bound form, each step involves detachment of the rear kinesin head from the MT, a forward move of the detached head, and its re-binding to the MT ahead of the other head. Steps are realized alternately by the two kinesin heads. Even though dynein also forms a homo-dimer, it does not exhibit this clear hand-over-hand movement[11]; of the two molecules, the same dynein molecule can move successively, which is not consistent with the hand-over-hand mechanism [10]. When one dynein subunit is mutated to inhibit the ATP hydrolysis reaction [10], even as dramatically as removing the ATPase module [16], the dimeric dynein still moves although with a modestly affected velocity. In contrast, kinesin motility is severely affected when one head is inactivated [17]. Although these facts exemplify the sharp differences in the motility of dynein from that of kinesin, the molecular mechanisms underlying dynein motility remain obscure.

**Fig 1:**
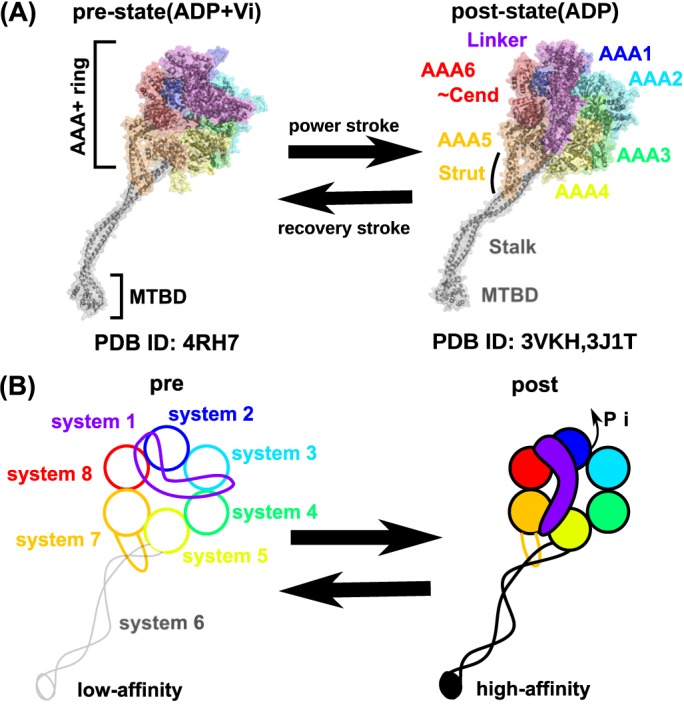
Structure of dynein motor domain. (A) Structure models for the pre-power-stroke structure (left, modeled from 4RH7, the protein data bank id) and the post-power-stroke structure (modeled from 3VKH and 3J1T). (B) Cartoon view of the two structures with the definition of the eight multiple-basin systems. A unified color code is used to distinguish the eight modules throughout this paper; from N-terminus, the linker is in purple, the AAA1 is in blue, the AAA2 is in cyan, the AAA3 is in green, the AAA4 is in yellow, the MTBD/Stalk is in grey, the AAA5 is in orange, and the AAA6/C-terminal modules is in red.

Cytoplasmic dynein is a giant protein complex containing two heavy chains, two intermediate chains, two light-intermediate chains, and six light chains. The C-terminal fragment of the heavy chain contains the motor domain comprising of an ATPase module and a MT-binding domain (MTBD). The overall architecture of the motor domain consists of a hexagonal AAA+ (ATPases associated with diverse cellular activities) ring that hydrolyzes ATP [18], the MTBD, and the Stalk which connects the AAA+ ring and the MTBD [19,20] (Fig 1A). Remarkably, the AAA+ ring is ~15 nm away from the MTBD. The affinity of the MTBD to MT is regulated via a nucleotide-dependent structural change in the AAA+ ring, suggesting long-distance allosteric couplings between the two elements. Understanding the mechanisms behind this long-range coupling was the focus in this study. Notably, this is a distinct structural feature, which differentiates dynein from kinesin where the core module hydrolyzes ATP and binds MT, directly coupling them in the same module.

The landmark X-ray crystallographic structure of *Dictyostelium discoideum* dynein-1 revealed a high-resolution structure of the full-length motor domain [21] (Fig 1A, right). The motor domain can be divided into eight distinct modules; six AAA+ modules making the hexagonal AAA+ ring (termed, in order of amino acid sequence, AAA1, AAA2, AAA3, AAA4, AAA5, and AAA6), the N-terminal linker which is docked on the planar surface of the AAA+ ring, and the Stalk/MTBD which protrudes from the AAA+ ring. At the C-terminal side of the AAA6, there is a C-terminal end segment, which is grouped with the AAA6 in this study. We refer to this module as the AAA6/C-terminal module. It should also be noted that the MTBD and the Stalk are inserted within the sequence of the AAA4 module. For the purposes of this study, we treated the MTBD and the Stalk together as a single module. At the junction between the AAA+ ring and the Stalk, a small element called the Strut/Buttress seems to augment/regulate the connection (Fig 1A). The Strut/Buttress sequence is embedded in the AAA5 sequence and thus is included in the AAA5 module in this study. Notably, even though the crystallographic diffraction indicates high resolution, the electron density near the MTBD is vague and the structural model for the MTBD region has low resolution. Only a combination of electron microscopy and computational modeling has provided a high resolution structural model of the MTBD with a high affinity for MT [22].

For the dynein motor function, nucleotide-dependent conformational changes have been partially characterized. Comparison of the above mentioned structure by Kon et al [21] with a recent crystallographic structure for human cytoplasmic dynein-2 by Schimidt et al [23] (Fig 1, left) reveals many structural changes, one of which is termed the “power-stroke” motion. In the pre-power-stroke state solved by Schmidt et al, the linker assumes a sharply bent form with its N-terminal tip approaching the AAA2 (Fig 1A left). Whereas in the post-power-stroke state solved by Kon et al, when looked from the side of the ring, the linker looks nearly straight so that its N-terminal tip extends to the AAA4 and AAA5 modules (Fig 1A right). The ATP- and ADP + Pi bound states are considered to form the pre-power-stroke state, while the ADP-bound and apo (nucleotide-free) states correspond to the post-power-stroke state [21,24–27]. Thus, the Pi-release event corresponds to the pre-to-post-power-stroke motions, while ATP binding couples the post-to-pre recovery-stroke.

Among the six AAA+ modules, the first four AAA+ modules possess ATP binding sites, in particular the AAA1 is known to play a major catalytic role in ATP-driven movement; a mutation that abolishes ATP hydrolysis in the AAA1 causes a complete loss of dynein motility [28,29]. The other ATP-binding modules appear to have regulatory roles [30–33].

The affinity of the MTBD for MT is regulated by a nucleotide-dependent conformational change. Generally, it is thought that the pre-power-stroke state corresponds to a weak-binding state of the MTBD, while the post-power-stroke state corresponds to a strong-binding state of the MTBD [34]. However, the mechanisms underlying this are unclear. For allosteric communication between the AAA+ ring and the MTBD, the Strut and the Stalk structures are considered to be important elements. The Strut move may be transmitted via the Stalk, which may then serve to change the structure of the MTBD [35].

In this study, we address allosteric couplings within the full-length motor domain of dynein-1, using a molecular simulation approach. Previously, molecular dynamics (MD) simulations have been used for dynein in structural modeling, fluctuation analysis, and a study of the interactions with the MT [22,36–39]. While highly desirable, the gold-standard atomistic MD simulation cannot be directly used at the moment for the current purpose due to the large size of the molecules and the high degree structural changes. To this end, we employed a coarse-grained MD approach where each amino acid is represented by one bead. For the last two decades, such coarse-grained MD approaches have been successfully employed to study a broad range of large-scale protein dynamics, such as folding, binding, and motor motions [40–45]. Notably, using the software CafeMol [46], we can utilize atomistic interaction information present in the reference X-ray structures even though we performed coarse-grained MD simulations. Using high efficiency computation, we conducted comprehensive simulations for both the pre-to-post-power-stroke transition and the post-to-pre recovery-stroke transition multiple-times (10–30 times per one setup) with multiple setups (35 setups in total). Assuming that the full-length motor domain is made of eight modules, we investigated the sequence of transitions in these eight modules, from which we reveal the allosteric couplings between these modules.

## Results

### Computational modeling of conformational change in dynein

To simulate large-scale conformational changes in dynein in a systematic manner, we employed a structure-based coarse-grained model where each amino acid is represented by a single bead located at the Cα atom. Starting with the atomistic models for the two reference structures, i.e. the pre- and post- power-stroke structures of dynein, we first estimated approximate pairwise residue interaction energies at atomic resolution. Feeding these energy values into the coarse-grained model, we set up an atomic interaction-based coarse-grained model (AICG2+ model) for both the pre- and post- power-stroke states [47,48]. Finally, integrating the AICG2+ model for the two-states, we constructed the multiple-basin model that empirically connects the two energy basins smoothly [49–51] (See also S1A Fig). Specifically, for dynein, in constructing the multiple-basin model, we assumed that conformational change can occur module by module. We assigned eight modules within the dynein full-length motor domain: (from N-terminal to C-terminal) the N-terminal linker, the AAA1, the AAA2, the AAA3, the AAA4, the MTBD together with the stalk region, the AAA5 including the Strut, and the AAA6-C-terminal module (Notably, since the MTBD is inserted within the AAA4 module, the AAA4 module contains two disconnected segments). For each module, together with its interactions to other modules, we set up one double-basin sub-system that connects the pre- and post- conformations (S1 Table and Fig 1B). Neighboring sub-systems interact with each other via module-interface interactions. Thus, in the simulation of the entire molecule, we observed sequential transitions for eight sub-systems, from which we analyzed conformational change pathways among, in principle, 8! conceivable orders of transitions.

We noted that, since the multiple-basin model is inherently empirical, we needed to introduce two parameters, Δ*V* for modulating the relative stability of the two states (pre- and post-) and *Δ* for deciding the height of the energy barrier between the two states for each module (S1A Fig). Through many preliminary simulations, we empirically tuned these parameters (See Method for details). Briefly, *Δ’*s were set so that the corresponding conformational transition occurs, on average, in the middle of the possible simulation time window. In contrast, Δ*V*‘s were tuned separately for power-stroke and recovery-stroke transitions so that we could observe the conformational transitions from the initial state to the target state. Although the tuned parameters were far from unique, we found that the allowed parameter ranges were rather narrow. We defined one set of these parameters that satisfied all the requirements as the standard set and these are listed in S2 and S3 Tables. Since the parameter choice was not unique, we varied Δ*V* values systematically (35 setups in total) repeating numerous simulations (10–30 trajectories for each setup), from which we sought the robust responses. We did not systematically change Δ value, because, by decreasing Δ significantly, we see virtually no structural change and, by increasing Δ, we often see too-frequent transitions or even appearance of an artificial minimum between the pre- and post- structures.

### Conformation change in the power-stroke: Overview

**Fig 2:**
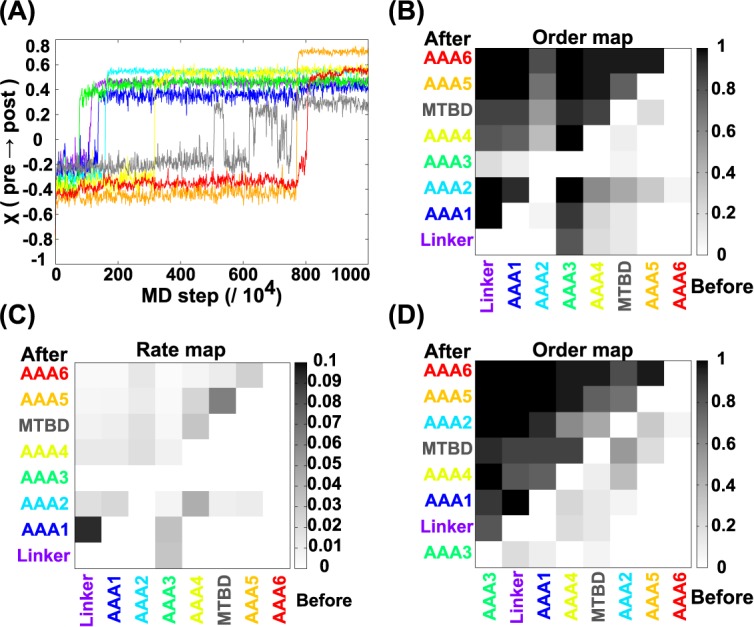
The power-stroke simulations in the standard setup. (A) A representative trajectory of the reaction coordinates χ for the eight modules along the MD step. (B) The order map. Each square represents the probability that the transition in the region in the horizontal axis occurred before that in the region in the vertical axis over 30 trajectories. (C) The pair rate map representing *k*(b|a). (D) The sorted order map. The data in (B) is rearranged so that the summation in each column is in descending order. The same color code is used as in Fig 1.

First, using the standard set of parameters, we performed molecular simulations of dynein from the pre-power-stroke state to the post-power-stroke state 30 times with different samples of stochastic forces. Fig 2A illustrates a typical trajectory of the reaction coordinates χ of all modules, which assume negative and positive values at the initial and final states, respectively (see METHODS for the definition of χ. See also S1 Movie). The trajectory in Fig 2A showed a representative trend in the order of transitions, although the order was to some extent stochastic in other trajectories. In Fig 2A, the AAA3 made the pre-to-post-transition first, which is followed by a change in the linker conformation. The linker conformational change triggers conformational changes in the AAA1 and subsequently in the AAA2. Following this, the transition cascade continues, in the order AAA4, MTBD, AAA5, and finally the AAA6/C-terminal module. Thus, with the exception of the first AAA3 transition, the order of change was sequential in sequence and occurred in a clockwise manner from a structural point of viewed from the side of the linker.

To statistically account for the order of the transitions of the modules, we calculated the probabilities, P-order (b ← a), that the transition in module “a” precedes that in “b” in 30 trajectory samples and the results are shown in Fig 2B (we refer to this map as an “order-map”). It should be noted that, when we observe multiple transitions between the pre and post states in a single trajectory, we use the time of the first transition for this analysis. In the figure, the dark square indicates that the module in the horizontal axis precedes the module in the vertical axis with a high probability (the *p*-value for the null hypothesis of the indistinguishable order of each pair of transitions is given in S2 Fig). For example, in the “linker” column, all rows except for AAA3 are dark, indicating that the linker conformational change occurs after AAA3, on average, and is before those of the other modules. The *p*-value for this particular order was 0.002 including the multiple testing correction so that we can clearly state that the AAA3 changes earlier than linker followed by the other transitions with statistical significance. Indeed, the order of transitions observed in Fig 2A was a representative case.

Generally, when a transition in one module occurs before a transition in another module, there can be two underlying mechanisms. In one, the two transitions are independent, but the characteristic time in one is shorter than that in the other (this is termed an “independent pair”). In the other mechanism, the transition in the first one is obligatory for the second transition (this is termed a “dependent pair”). We distinguished the two cases by the following two analyses. First, to infer whether a pair of transitions is independent or dependent, we calculated the average pair rate constants *k*(b|a), for the transition in “b” after the transition of “a”, defined as $k(b|a)=(1/n)\sum_{i=1}^{n}1/\Delta t_{ab}(i)$ where the summation is taken over the trajectories in which “b” transited later than “a”. Δ*t*_*ab*_(*i*) is the time interval from the transition in a to that in b in the *i*-th trajectory, and *n* is the number of the corresponding trajectories. When transition “b” did not occur, we set the time of the “b” transition as the final time of the simulation. Fig 2C shows the pairwise rates *k*(b|a) for the standard set, in which the value of *k*(b|a) is represented by the darkness (empty if *n* < 7). Thus, the dark squares in the plot suggest that these pairs are “dependent pairs”. Additionally, we also performed bootstrapping (S3 Fig). These dependent pairs are discussed in more detail in subsequent sub-sections.

As an additional analysis of the dependence, we consider a hypothetical and perfectly-independent pair of systems, A and B, both of which make transitions with single-exponential time courses. The rate constants for A and B were set as those obtained from the above MD simulations. Then, for the perfectly-independent system, we calculated the analytical probability density function of the difference in the time of transitions of the two modules A and B (“null” hypothesis). This distribution can directly be compared with the time difference histogram obtained from the actual MD simulations (S4 Fig). The difference between the hypothetical probability distribution (“null” hypothesis) and the observed histogram was assessed by Kolmogorov-Smirnov test (S4 Table).

By sorting the eight modules in descending order of P_i_ = Σ_j_ P-order (*j* ← *i*) we obtained an average order of transitions, as shown in Fig 2D. Notably, for many pairs of modules, the orders of transitions were fully deterministic (i.e.; the probabilities were zero or one), whereas, for some pairs, the orders of transitions were stochastic. Moreover, some orders of transitions depended heavily on the parameters Δ*V* and Δ. Hereafter, we focused on “robust” orders of transitions that were found in the majority of trajectories and were not sensitive to changes in the parameters.

### The power-stroke pathway: The linker transition precedes the AAA1 transition

In the standard setup, the linker transition, i.e., the linker power-stroke, occurred before the AAA1 transition with a probability of one (P-order (AAA1 ← linker) = 1.0). In most trajectories, the AAA1 transition occurred immediately after the conformational change in the linker: The pairwise rate *k*(AAA1|linker) = 0.093 (95% confidence interval [0.065, 0.13], S3A Fig) (hereafter, the rate is given in the unit 1/10^4^ MD steps) is large, as shown in the map in Fig 2C. Importantly, the transition in the AAA1 is the nucleotide-dependent process corresponding to the release of phosphate from the AAA1 module in the power-stroke process. The current result that the linker movement is a prerequisite for the AAA1 transition implies that the transition in the AAA1 module needs to be unlocked by the thermally-activated spontaneous linker movement.

**Fig 3:**
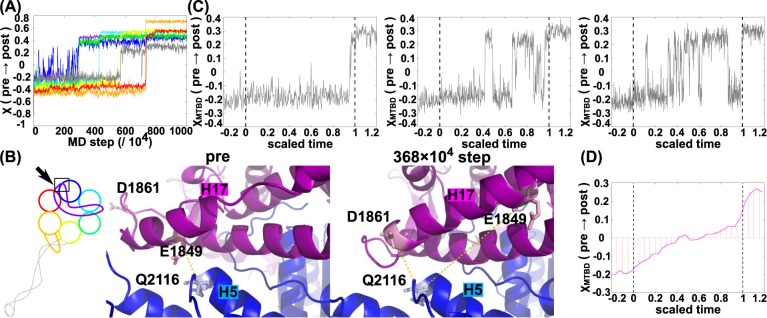
Analysis of the power-stroke process. (A) A representative trajectory for the AAA1↑ simulation. (B) Local structural change at the interface between the linker and AAA1 in the standard setup simulation. The middle and the right structures represent the pre-power-stroke and an intermediate snapshot structure, respectively. The left-hand cartoon indicates the position of the structure within the entire motor domain. Important interactions are indicated by dashed lines. Atomistic model was reconstructed by the protocol given in Method. (C) Three examples of the conformational change in MTBD/Stalk along scaled time. (D) Average conformational change in MTBD/Stalk along scaled time for power-strokes. The time in each trajectory is scaled.

To test the mechanical dependence of the two transitions, we examined the following two cases. First, we examined the effect of retarding the linker conformational change (denoted as “linker ↓”). The retarding effect can easily be introduced by increasing the Δ*V* value for the corresponding module (S2 Table). By introducing this change, we did indeed observe a delay in the timing at which the linker made its conformational change. Importantly, we found that AAA1 did not make its transition before the linker conformational change and this was true in all trajectories examined (P-order (AAA1 ← linker) = 1.0) (Refer to setup 2 in S5 Fig which shows all of order map data obtained from power-stroke simulations). Second, we examined the effect of accelerating the AAA1 conformational change (denoted as “AAA1 ↑"). Under this condition, we did not observe a significant change in the order of transitions; the linker conformational change almost always preceded the AAA1 transition (P-order (AAA1 ← linker) = 0.8, while P-order (linker ← AAA1) = 0.1) (see Fig 3A for a representative trajectory). We also performed another statistical test supporting these dependences (S4A Fig, S4 Table). Taken together, these data suggest that the conformational transition in AAA1 is dependent on the linker being in the post-stroke conformation.

Fig 3B depicts the major interaction interface between the linker (purple) and the AAA1 module (blue) in the pre-stroke state (left) and in an intermediate snapshot (after the transition of the linker) (right). The figure also include some important residue-residue interaction, selected based on its large change between the pre- and post- structures, primarily electrostatic interactions, and relatively high sequence conservation (a multiple sequence alignment given in S6A Fig). In the pre-stroke state, a major interaction occurs between E1849 in the linker H17 helix and Q2116 in the loop between the H5 and H6 helices of AAA1. This interaction is lost in the intermediate and the post-stroke states. Instead, a contact between D1861 in H17 in the linker and Q2116 is observed in the intermediate and the post-stroke states. The dissociation of E1849 from Q2116 may be responsible for unlocking the conformational transition in the AAA1. We did not observe any marked structural changes in the N-terminal loop of AAA1, which connects the AAA1 with the linker.

Technically, since we included the interaction energies between the linker and AAA1 into the linker multiple-basin system (S1 Table), this could, in principle, have affected the order of transitions. To check this possibility, we performed a control set of simulations in which the interaction between the linker and AAA1 is included into the AAA1 multiple-basin system. The result shown in S1C Fig is essentially unchanged from the standard setup. Thus, the result is not affected by the particular simulation setting.

### The power-stroke pathway: The middle stages

In all the trajectories in the standard setup, the AAA4 module state transition occurred after the AAA3 module conformational change (Fig 2B, P-order (AAA4 ← AAA3) = 1.0). To test the robustness of this order of events, we examined the effect of retarding the AAA3 conformational change (referred to as "AAA3 ↓"). The results demonstrated that the AAA3 module still changed its conformation before the AAA4 module changed its (P-order (AAA4 ← AAA3) = 0.9 and P-order (AAA3 ← AAA4) = 0.0). Next, we examined the effect of accelerating the AAA4 conformational change (referred to as "AAA4 ↑"). Even in this case, the AAA3 made the transition earlier than the AAA4 with the probability one. However, the pair rates were modest in both directions. In the setup, AAA3 ↓, *k*(AAA4|AAA3) = 0.019 (see setup 6 in S7 Fig), while in the setup AAA4 ↑, *k*(AAA4|AAA3) = 0.047 (in comparison the fast response *k*(AAA1|linker) = 0.093 in the standard setup (95% confidence interval [0.0060, 0.011], S3B Fig)). Thus, the allosteric coupling between the two transitions appears to be modest. Taken together, these data suggest that the conformational transition in the AAA3 promotes the conformational change in the AAA4, although the coupling is not as strong as that between the linker and the AAA1 (S4B Fig, S4 Table). We note that the summation of P-order does not always equal to 1 due to the trajectory where one of the two modules did not make transition at all until the end.

Transitions in the AAA2 were uncoupled from other transitions in the current simulations. By retarding or accelerating the AAA2 transition, we observed the corresponding changes in the order of AAA2 transition and noted that these change did not have a clear effect on other transitions. These data suggest that the AAA2 transition does not play a crucial role in the power-stroke motion, which is in agreement with the experimental result that inhibition of ATP/ADP binding to the AAA2 module does not affect the motility of dynein [31].

From both functional and structural perspectives, the most important allosteric coupling must be the conformational transition order between the MTBD and the AAA+ ring, i.e., which occurs earlier. In most trajectories, the MTBD stayed in the pre-stroke state before the AAA4 transition, and then showed stochastic and reversible transitions between the pre- and post- stroke states after the AAA4 module transition (see for example Fig 2A). When the AAA5 module transitioned to its post-stroke state, the MTBD conformation was fixed in its post-stroke state. To demonstrate this behavior systematically, we used a scaled time, (scaled-time = (*MDstep* − *τ*_*AAA*4_)/(*τ*_*AAA*5_ − *τ*_*AAA*4_), where *τ*_*AAA*4_ and *τ*_*AAA*5_ are the MD time steps of the AAA4 and AAA5 module transitions in each trajectory, respectively). The χ value for the MTBD was plotted against scaled-time for three additional trajectories in Fig 3C. These data suggest that the MTBD (including the Stalk) transition depends on both the AAA4 and AAA5 states. Naturally, this might be expected since the Stalk is inserted in the AAA4, and the Strut in the AAA5 interacts with the Stalk, thereby moving the Stalk (referred to as the ‘open-zipper’ model, see also Fig 1A). We also plotted the χ value for the MTBD averaged over 30 trajectories against scaled time in Fig 3D, showing that χ started to increase upon the AAA4 transition and peaking after the AAA5 transition. These data imply that the AAA5 module has a somewhat larger effect on the MTBD conformation than the AAA4 module.

### The power-stroke pathway: The last step is the AAA6/C-terminal module transition

In the majority of trajectories, the last part of the transition cascade consisted of the transition of the AAA5 followed by that of the AAA6/C-terminal module. This final pair of steps was very robust. Even when a retardation in the AAA5 transition was introduced (referred to as "AAA5 ↓")), the order of transitions did not change (P-order (AAA6 ← AAA5) = 0.5 and P-order (AAA5 ← AAA6) = 0.0). Moreover, even when we introduced an acceleration of the AAA6/C-terminal module transition (referred to as “AAA6 ↑"), the order of the two events was completely unaffected (P-order (AAA6 ← AAA5) = 1.0, P-order (AAA5 ← AAA6) = 0.0).

### Conformation change in the recovery-stroke: Overview

**Fig 4:**
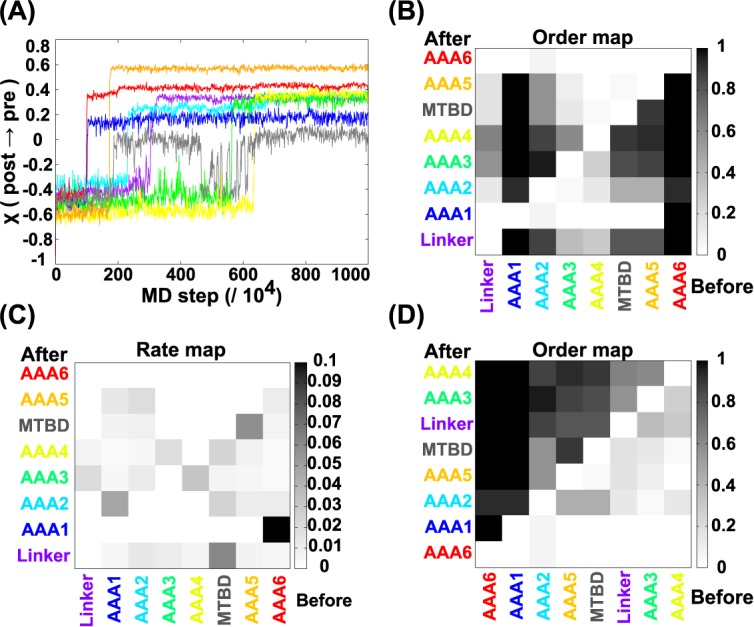
Recovery-stroke simulations in the standard setup. (A) A representative trajectory of the reaction coordinates χ for the eight modules along the MD step. (B) The order map. (C) The pair-rate map. (D) The sorted order map. Refer to the caption for Fig 2 for more information.

Next, we investigated the pathways for the recovery-stroke from the post- to pre-power-stroke states using the standard parameter set for this process. Fig 4A depicts a representative trajectory, where the transition occurred in the order of the AAA6/C-terminal module, the AAA1, the AAA5, the MTBD, the AAA2, the linker region, the AAA3, and finally the AAA4 (S2 Movie). As the average over 30 trajectories, the order-map for this recovery processes (see Fig 4B for the original index order and Fig 4D for the optimally sorted module order) supports the same order of transitions, on average, as those in Fig 4A. Structurally, this order of transitions contains two parallel cascades, both initiated from the AAA6/C-terminal module. In one pathway, the transition in the AAA6/C-terminal module propagated to that in the AAA5 and then to the MTBD (anti-clockwise in the viewing angle of Fig 1). In the other pathway that proceeds in the opposite orientation, the transition in the AAA6/C-terminal module propagated to transitions in the AAA1, the AAA2, the linker, the AAA3, and finally the AAA4 (clockwise). Of note, the AAA2 module is markedly independent of the other modules, meaning that the AAA2 can make transitions largely independent of other structural changes. The rate map in Fig 4C points out a strong dependency; namely that the AAA6/C-terminal module transition is immediately followed by the AAA1 transition.

### The recovery pathway: The first step is the transition in the AAA6/C-terminal module

In nearly all of the trajectories in the recovery-stroke simulations, we observed that the conformational change cascade is initiated by the transition in the AAA6/C-terminal module.

**Fig 5:**
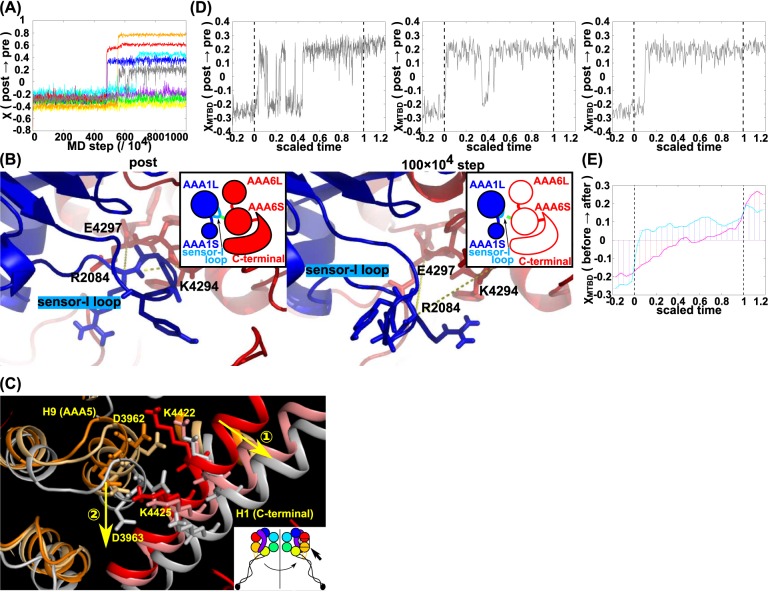
Analysis of the recovery-stroke process. (A) A representative trajectory for the AAA6↓ simulation. (B) Local structural change at the interface between AAA6/C-terminal module and the AAA1 in the standard setup simulation. The post-power-stroke structure (left) and an intermediate snapshot structure (right) are depicted. For the latter, the atomic structure was reconstituted from a coarse-grained snapshot. E4297 in the AAA6/C-terminal module and R2084 in the AAA1 module interact in the post-power-stroke structure, whereas they are apart in the intermediate structure. Atomistic model was reconstructed by the protocol given in Method. (C) Local structural change at the interface between the AAA6/C-terminal module (red for the post-power-stroke structure), salmon pink (100 x 10^4^ MD step), and white (103 x 10^4^ MD step)) and the AAA5 module (orange for the post-power-stroke structure), light orange (100 x 10^4^ MD step), and white (103 x 10^4^ MD step) in the standard setup simulation. Arrows labeled with 1 and 2 represent the sequential movement. (D) Three examples of the conformational change in MTBD/Stalk along scaled time. (E) Averaged conformational change in the MTBD/Stalk along scaled time for the forward (red) and recovery (blue) strokes. The time in each trajectory is scaled to be zero at the time of the AAA4 (AAA5) transitions and one at the time of the AAA5 (AAA4) transitions for the forward (recovery) strokes.

When we retarded the transition in the AAA6/C-terminal module, (referred to as “AAA6 ↓") we found that the order of transition events did not change (P-order (AAA6 ← AAA5) = 0), and that all of the subsequent events were retarded (see setup 2 in S8 Fig). Fig 5A shows that other modules did not make their transitions until AAA6/C-terminal module transited. As a result, due to this delay in all transitions, some modules did not complete their transitions within the simulation time. This result may be correlated to experimental data showing that a C-terminal truncated mouse dynein shows markedly larger stall-force compared to wild-type dynein [52]. This may suggest that the truncated dynein lacking the C-terminus tends to stay in its post-stroke state maintaining a strong affinity for tubulin.

### The recovery pathway: The conformational change in the AAA6/C-terminal module induces the AAA1 and AAA5 transitions

In the case of standard parameter set, as shown in Fig 4, the AAA1 module made the transition immediately after the transition in the AAA6/C-terminal module. Even when we introduced an accelerating effect in the AAA1 transition (referred to as “AAA1 ↑) the order of the pair of events did not change. (P-order (AAA1←AAA6) = 1.0 and P-order (AAA6←AAA1) = 0.0). Thus, this order is strictly adhered to. This dependence of the AAA1 transition on the conformational change of the AAA6/C-terminal module suggests that the ATP hydrolysis cycle in the AAA1 module is tightly regulated by the AAA6/C-terminal module conformation at the time of the recovery-stroke. The result suggests that thermally-activated transition in the AAA6/C-terminal unlocks the nucleotide-dependent AAA1 transition. In the post-stroke structure, the interface between the AAA1 and the AAA6 contains the salt bridge interaction between R2084 in the sensor-I loop in AAA1 and E4297 in the AAA6 module (Fig 5B left). In the simulation, when the AAA6/C-terminal module made the transition to the pre-stroke conformation, this salt bridge is lost and the interface became loose (Fig 5B right), which allows the sensor-I loop to move significantly, triggering the conformational transition in the AAA1 module. In the pre-stroke structure, the sensor-I loop in the AAA1 module reaches to the interface with the AAA2 module enabling the sensor-I motif (N2078) to contribute to ATP binding (see S6B for the sequence conservation of this site).

The AAA5 module transition occurred after those of the AAA6 and AAA1 modules. However, the AAA1 does not interact directly with the AAA5 and thus is unlikely to trigger the AAA5 transition itself. Instead, most likely, the AAA5 transition is triggered by the AAA6 transition via the interface. As above, when the AAA6/C-terminal module transition was retarded, the AAA5 did not make its transition. Conversely, when the conformational change in the AAA5 was accelerated (referred to as “AAA5 ↑"), we found that, in some cases, the AAA5 conformational change preceded that of the AAA6/C-terminal module (P-order(AAA6←AAA5) = 0.3). Yet, in these cases, the conformational change in the AAA6/C-terminal module almost immediately followed that of AAA5. Thus, these data may also support the idea that other modules do not readily change their conformation prior to the change in the AAA6/C-terminal module.

To understand the allosteric propagation from the AAA6/C-terminal module to the AAA5 module, we examined the interface between the C-terminal module and AAA5 during simulations (refer to Fig 5C where AAA5 is shown in orange and the AAA6/C-terminal module in red). In the early stage of the trajectories from the post-power-stroke structure, a long α helix (H1 helix) in the C-terminal module moved away from the AAA5 module (red to pink, indicated by an arrow with 1 in a circle). This H1 helix contains K4422 and K4425, which make salt bridges with D3962 and D3963, respectively, in the post-power-stroke structure (these sites are well conserved as in S6C Fig). As a result of this movement of the H1 helix, these interactions are lost. Later on, the H9 helix in the AAA5 module containing D3962 and D3963 moved further (indicated by an arrow with 2 in a circle). One end of the H9 helix (not depicted in Fig 5C) interacts with the C-terminal end of Strut so that movement of this helix transmits the change into Strut. The Strut shift with respect to Stalk affects the MTBD conformation.

### The recovery pathway: The AAA2 transition weakly promotes recovery-stroke of the linker

The conformational change in the AAA2 was observed after the transition in the AAA1 (Fig 4). To test this dependence, we introduced a retardation in the transition of the AAA1, (referred to as “AAA1 ↓”), which, in many cases, resulted in a change in the order of transitions between the AAA1 and AAA2 modules (P-order (AAA2←AAA1) = 0.6 and P-order(AAA1←AAA2) = 0.4). Next, we accelerated the transition of the AAA2 (referred to as “AAA2 ↑”), which resulted in a change of transition order; in half of the trajectories, the AAA2 changed its conformation prior to that of the AAA1. In these trajectories, the AAA2 transition occurred even earlier than that of the AAA6/C-terminal module. These results suggest that the AAA2 transition does not depend on other transitions. In the standard setup, the AAA1 module transited rather quickly after the AAA6/C-terminal module transition, while the transition of the AAA2 is inherently slower.

Next, we investigated the role of the AAA2 module transition on subsequent processes, by introducing a retardation in the AAA2 transition (referred to as “AAA2 ↓”). The data revealed a weak correlation between the AAA2 module transition with that of the linker (*k*(linker|AAA2) = 0.020, P-order (linker←AAA2) = 0.6, P-order (AAA2←linker) = 0.2) (refer to setup 12 in S9 Fig). We also accelerated the transition of the linker, (referred to as “linker↑”), which in most cases did not result in a change in the order of transitions (P-order (linker←AAA2) = 0.8, P-order (AAA2←linker) = 0.2). Moreover, the pair transition rate increased from that seen in the standard setup (*k*(linker|AAA2) = 0.013 (95% confidence interval [0.0041, 0.023], S3C Fig)). These data suggest a weak dependence of the linker recovery-stroke motion on the AAA2 transition. Another statistical test supported these observations (S4C Fig, S4 Table).

Next, we investigated the correlation between the AAA2 and AAA3 module transitions. In the “AAA2 ↓” simulations, we found a change in the order of transitions in two trajectories out of ten, (P-order (AAA3←AAA2) = 0.5, and P-order (AAA2←AAA3) = 0.2). When we accelerated the AAA3 transition (referred to as “AAA3↑”), the order of transitions did not change at all. However, their pair rates were small, *k*(AAA3|AAA2) = 0.023 in the AAA3↑ setup, and *k*(AAA3|AAA2) = 0.011 (95% confidence interval [0.0044, 0.018], S3D Fig) in the standard setup (Fig 4C). Thus, we conclude that the AAA3 transition is only weakly dependent on the AAA2 transition (see also S4D Fig and S4 Table).

### The AAA5 transition supports structural changes in the MTBD

Finally, we addressed the structural change in the MTBD, directly related to the dynein affinity for the MT. Fig 5D depicts three additional trajectories of MTBD motions along scaled time (defined as scaled-time = (*MDstep* − *τ*_*AAA*5_)/(*τ*_*AAA*4_ − *τ*_*AAA*5_)) in a manner similar to that in the power-stroke case; the scaled time is zero at the time of the AAA5 transition, and is one at the time of the AAA4 transition. Fig 5D shows that the AAA5 transition immediately triggers a high degree of fluctuation in MTBD between the pre- and post- states. These repetitive transitions ceased when the AAA4 module made its transition to the pre-stroke state. Thus, as was the case for the power-stroke, the MTBD motions in the recovery-stroke are determined by its two neighboring modules namely, AAA4 and AAA5.

To address which of the AAA4 and AAA5 modules play a dominant role in the MTBD transition, we plotted the averaged reaction coordinate, χ_MTBD,_ against the scaled time both for the power-stroke and the recovery-stroke motions in the standard setup (Fig 5E). Here, the average was taken over 30 trajectories for each of the cases. We noted that χ _MTBD_ moved more rapidly for the recovery-stroke case than for the power-stroke case. Of note, the scaled times zero and one correspond to the time of the AAA5 transition and that of the AAA4 transition in the recovery-stroke, respectively. On the other hand, over the same time range in the power-stroke case, the scaled times zero and one coincide with the time of the AAA4 and AAA5 transitions, respectively (Fig 3D). This implies that, of the two, the AAA5 module seems to have a stronger effect on the motion of the MTBD.

## Discussion

### Allosteric conformational change cascade

**Fig 6:**
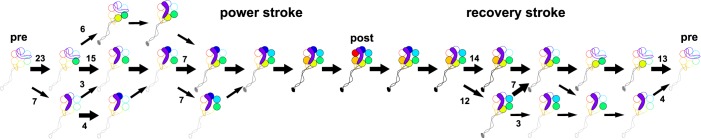
Summary of the conformational change cascade in the power-stroke (left-half) and the recovery-stroke (right-half) processes. The open and filled symbols represent the pre- and post-power-stroke states, respectively. Only for the MTBD, we used a three-state representation with the shaded circles being in a highly fluctuating state. The thickness of arrows indicates probabilities. The numbers associated with arrows represents the numbers of trajectories. Linker in purple, AAA1 in blue, AAA2 in cyan, AAA3 in green, AAA4 in yellow, stalk~MTBD in grey, AAA5 in orange, and AAA6~C-sequence in red.

We identify the major allosteric transitions that occur in cytoplasmic dynein during the power-stroke and the recovery-stroke motions. Using the standard set up and a statistical analysis of the order-map, we sorted the modules in the rows and the columns so that the summation of the probabilities in each column is in descending order (Fig 2D for the power-stroke and Fig 4D for the recovery-stroke). The resulting sequences in the rows/columns represent the dominant pathways that occur during the power-stroke and the recovery-stroke motions. Including sub-dominant pathways, we constructed a network of allosteric transitions for the power-stroke and recovery-stroke transitions as shown in Fig 6.

In the power-stroke motion, the AAA3 transition precedes the linker transition in the standard setup, although this order is not obligatory in other setups. From a structural perspective, we see some interactions between the tip of the linker (helices H6 and H7) and a loop (called “pre-Walker B”) in the AAA3 in the pre-power-stroke state (S10 Fig), which may serve as a hook. Once these contacts are lost in the intermediate snapshot (S10 Fig), the linker motions are activated. However, as noted above, the precedence of the AAA3 transition is not a robust property in simulations and thus is not to be emphasized (S6D Fig).

Then, the critical conformational transition occurred in the linker, which is immediately followed by the AAA1 transition. This order was very robust, being observed in any of the setups we applied. The nucleotide-dependent AAA1 transition may be unlocked by the precedent linker movement. Subsequently, the AAA4 module transition occurred. The transition of the AAA2 was stochastic and only weakly related to the other transitions. On average, the AAA2 transition occurred either before or after the AAA4 transition (Fig 2D). The transition cascade of linker, AAA1, AAA2, AAA3, and AAA4 constitutes the first half of the transition in the power-stroke process. The MTBD, including the Stalk, started to exhibit large-scale fluctuations after the transition of the AAA4. However, it took some time for this module to adopt its post-power-stroke structure. After this time interval had passed, the second half of the transition started with the AAA5 transition. The AAA5 transition appears to be dependent on the preceding transitions of the AAA4 and the linker via direct contacts in the post-power-stroke structure (S5 Table) [21]. Once the AAA5 module assumed its post-power-stroke structure, the Strut loop in the AAA5 stabilized the Stalk and MTBD in their post-power-stroke structure. Finally, the AAA5 transition triggered the AAA6~C-terminal module transition, which robustly terminated the whole process. Overall, the transition cascade proceeded in a clockwise manner looking from the linker attached side.

In the recovery-stroke motions, the first transition to occur was that of the AAA6/C-terminal module. This order was very robust. In fact, no other module could make a conformational change before the AAA6/C-terminal module transition. The AAA6/C-terminal module transition unlocked a cascade of transitions in two directions on the AAA+ ring. In one, the AAA6/C-terminal module transition enabled the AAA1 module to make the nucleotide-dependent transition, which was followed by the AAA2 module and the linker transition (i.e. the clockwise direction). In the other direction, the AAA6/C-terminal module triggered the AAA5 transition, which was followed by the AAA4 and MTBD transitions (i.e. the anti-clockwise direction). The timing of the AAA3 transition was stochastic occurring either after the linker recovery or after the AAA4 transition. This is reasonable since the AAA3 module has direct contacts with both the AAA4 module and the linker (when the linker takes the pre-power-stroke structure). Recent experiments have shown that ATP hydrolysis in the AAA3 module stabilizes the weak-binding state of the MTBD [53]. The current simulation suggested that the state of the MTBD is largely regulated by the state of the AAA5 module. Taken together these data imply that for the AAA3 transition to affect the transition of the MTBD, the AAA3 module transition has to precede the AAA5 module transition. However, in our simulation this was not realized in most cases. This is probably because we used a pair of crystallographic information where the nucleotide state is different only in the AAA1 module, and thus the effect of ATP hydrolysis in the AAA3 module could not be reproduced. The same experimental work [53] showed that hydrolysis in the AAA3 module is not well correlated with the hydrolysis cycle in the AAA1 module, which is in accord with the current work where the AAA3 module transition shows little correlation with the AAA1 module transition in the power-stroke motion.

As a sub-dominant pathway of the recovery stroke process, we observed the case where the linker recovery stroke motion occurred in the last step (drawn as the lower branch path in Fig 6). This pathway well correlates with a scenario that the linker movement is induced by the steric contact of the N-terminal tip of the linker with PS-I insert of the AAA4 large sub-module [23]. We note, however, that this pathway was sub-dominant observed in 4 cases out of 17 complete recovery-stroke trajectories. The scenario is motivated by the structural insight and is supported by the experimental data that the mutation in AAA4 reduces the dynein motility [23]. Notably, in our dominant pathway (13 out of 17, drawn as the upper path in Fig 6), the structural stability of MTBD/Stalk well depends on AAA4 so that it is also compatible with the same experiment.

It should be emphasized that the power-stroke and recovery-stroke allosteric pathways are not merely the reverse order of each transition. The power-stroke allosteric transitions in the AAA+ ring occurred in a clockwise manner starting from the linker/AAA1 modules and ending at the AAA6/C-terminal module transition. The recovery-stroke started with the AAA6/C-terminal module transition, which is indeed opposite to the power-stroke step. However, the recovery allosteric transitions then propagated in a bidirectional manner, both clockwise and anti-clockwise. This asymmetry might have important functional implications for the uni-directional movement of dynein.

### Relevance to motility

To understand dynein motility from a structural perspective, it is important to understand the status of the MTBD when the linker makes its conformational changes. If the MTBD is in a high-affinity state for the MT at the timing of the linker transition, dynein transmits the linker stroke directly to the MT. Otherwise the linker stroke does not provide a strong force to the MT.

In the power-stroke process, our simulation clearly predicts that the MTBD is in the low-affinity state for MT (i.e., the pre-power-stroke state) when the linker makes its conformational change. This is apparently in sharp contrast to the conventional model for dynein motility, in which the MTBD is assumed to take a high-affinity state for MT when the linker makes power-stroke swing [54]. Here, we discuss two points. 1) For dynein, there is no direct evidence, to our knowledge, for this conventional assumption. Instead, this assumption is based on its analogy to other linear dimeric molecular motors, kinesin and myosin. It should be noted that the motility of dynein is markedly different from those in kinesin and myosin. Thus, the analogy is not necessarily true. There are some evidences that show marked differences in dynein motility from kinesin motility. For example, dynein still proceed along MT even if one motor domain is replaced with a simple stick-like crutch [16]. Thus, the assumption that MTBD has a high affinity to the MT at the timing of power stroke has no support in dynein. 2) From structural perspectives, the linker is next to the AAA1 module where ATP hydrolysis drives the conformational transitions. Conversely, the MTBD is far away from the AAA1. If the conformational changes propagate through physical contacts at their interfaces, it is unlikely that the MTBD makes the transition earlier than that of the linker. Our simulation results are reasonable in that the conformational change cascade went through physical contacts, which resulted in the preceding transition of the linker with the MTBD in its low-affinity state for MT. However, we note that experimental data unambiguously show that the linker power-stroke motion is necessary for the dynein motility [55]. We consider two possible explanations. First, the linker may contribute to the stability of the AAA+ ring, which contains marked gaps between AAA+ modules. Secondly, if the linker power-stroke occurs while the MTBD being in the low-affinity state, the AAA+ ring is displaced or rotated with respect to the other monomer, which could induce the MTBD sliding towards the minus end of MT. After the sliding, the MTBD may changes its conformation to the high-affinity state for MT. Together, the dynein may achieve a move towards minus end of MT. In this model too, the linker power-stroke is an indispensable step, making it compatible with the experimental data.

For the recovery-stroke, the situation is subtle. Starting from the AAA6/C-terminal conformational change, the clockwise cascade of transitions induces the linker conformational change, while the MTBD/Stalk structure change is a part of the anti-clockwise propagation from AAA6. Thus, the linker and the MTBD structural changes are not directly linked mechanistically so that their timings are not clearly predicted in the simulations. In the standard setup, P-order (MTBD←linker) = 0.167 and P-order (linker←MTBD) = 0.8, indicating that the MTBD tends to make transitions earlier, on average (Fig 4B). When we introduced an acceleration in the linker transition (linker↑), the order of transitions was P-order (MTBD←linker) = 0.6 and P-order (linker←MTBD) = 0.4. These data suggest that the order of these two events is not robust and thus is not well controlled in the current simulations.

We note that the conformational change cascades given in Fig 6 are regarded as theoretical prediction, which needs to be verified experimentally. In principle, these can be examined by multicolor FRET for example. With regard to Fig 5C, by introducing the deletion mutant of the C-terminal helix 1, we can test how much that Helix 1 contributes to structure determination of MTBD over Strut in AAA5.

### Limitations in the current work

While the current study provided a detailed description of the allosteric conformational changes that occur in dynein, it has some limitations. Since we relied on structure-based MD simulations, the result is largely dependent on the given pair of reference structures. First, the pre-power-stroke structure was obtained for human dynein-2, which differs in sequence and function from *Dictyostelium discoideum* dynein-1 for which the post-power-stroke structure is available (Identity is 28%). Second, in both of the reference structures, the AAA2, AAA3, and AAA4 modules have ADP bound. Therefore with these structures alone, we cannot address the role of ATP hydrolysis in these three modules. Once a high-resolution structure is obtained for the different nucleotide-bound forms of these modules, we can use them to address this point. Third, related to the first point, because the sequence is less conserved in the C-terminal module, we could not accurately model the *Dictyostelium discoideum* dynein-1 structure from the human dynein-2 C-terminal module. This precluded us from treating the C-terminal module as a separate unit of transition. It was desirable, but not possible, to treat the C-terminal module independently from the AAA6/C-terminal module. Fourth, the multiple-basin model contained the parameters, *Δ* and Δ*V*, to smoothly connect the two reference structures. These parameters were treated in this work as empirical parameters tuned to realize conformational changes within the simulation time. We can, in principle, determine these parameters based on either experimental data, if available, or atomistic molecular simulations. Finally, to understand the motility of dynein on the MT, ultimately we need to simulate the dynein-MT complex. Moreover, since the method used in this work is very general, we can apply the same approach to study other multi-domain proteins.

## Methods

### Proteins

The simulated protein was *Dictyostelium discoideum* cytoplasmic dynein-1 (UniProtKB (P34036). For modeling/analysis, we divide the motor domain Q1522-I4730 into eight structural units; the linker (Q1522~Y1935), AAA1 (Y1936~Q2229), AAA2 (P2230~V2631), AAA3 (P2632~R2948), AAA4 (P2949~D3262, A3597~L3638), Stalk~MTBD (F3263~S3596), AAA5 (S3639~Q4114), and AAA6/C-terminal end (E4115~I4730) (Fig 1). Of note, the AAA4 module contains two disconnected segments, between which the Stalk~MTBD is inserted.

### Structural modeling

For the reference structure of the post-power-stroke state, we primarily used the X-ray crystal structure of *Dictyostelium discoideum* cytoplasmic dynein in the ADP-bound form (pdb ID: 3VKH)[21]. This structural model has low resolution near the MTBD region. Thus, for the MTBD region, we used a model obtained by a combination of cryo-electron microscopy and molecular simulations for *Mus musculus* cytoplasmic dynein (pdb ID: 3J1T) [22]. Clustal ω was used to align the two reference sequences [56,57]. MODELLER was used to make a homology model and augment missing residues [58]. Using CafeMol 2.1 [46], we first converted the Cartesian coordinates of the two reference structures into internal coordinates. Combining all but the MTBD coordinates from 3VKH and the MTBD (V3350~K3514 in *Dictyostelium discoideum* and K3264~K3427 in *Mus musculus*) coordinates from 3J1T, we obtained a hybrid structure in the internal coordinate representation. Using CafeMol 2.1 with this hybrid coordinate as the reference, we performed a very short simulated annealing simulation (essentially, minimization) to reach a stable three dimensional structure with the coarse-grained representation. PD2 ca2main [59] and Scwrl4 [60] were used to reconstruct the atomic model for the backbone and the sidechain, respectively. This reconstituted atomic structure was used as the reference structure of the post-stroke state. To test the modeled structure, we checked the local stability of the model by performing short atomistic MD simulations with GROMACS 5.1.1 [61,62].

For the pre-power-stroke structure, the full-length motor domain structure is solved only for human dynein-2 in the ADP + Pi bound form (pdb ID: 4RH7) [23], in which Q1255~V4308 was utilized as the template. Using this as the template, we obtained a homology model for *Dictyostelium discoideum* cytoplasmic dynein-1 using MODELLER 9.15. In the same manner as described above for the pre-power-stroke state, after a short simulated annealing run with CafeMol, we used PD2 ca2main, and subsequently Scwrl4, to reconstruct an atomistic structure. This modeled atomic structure was used as the reference structure of the pre-power-stroke state. To test this structure, it was subjected to MD simulations with GROMACS 5.1.1, confirming that the modeled structure is locally stable.

In the MD simulation using GROMACS, we used the amber99sb-ildn force field for dynein [63] and TIP3P for solvent water [64]. We added sodium and chloride ions to neutralize the system and to make the salt concentration approximately equal to 0.1 M, which resulted in 668 sodium ions and 627 chloride ions in 322,307 (283,925) water molecules in the pre-power-stroke (post-power-stroke) conformations. The energy minimization by the steepest descent minimization algorithm was followed by equilibration of solvents with NVT and subsequently NPT ensemble simulations for 100 ps at 300 K. In the production run, we used NPT ensemble with 1 atm and 300 K. Long range electrostatics were calculated by the Particle-Mesh-Ewald method. The production run was conducted with 1 fs step for 10 ns. It should be noted that the purpose of this atomistic MD simulation was to simply check the local quality of the model. The RMSD from the initial structure (S11 Fig) shows saturation around 0.5 nm, which, considering the large size of AAA+ ring, we regard it reasonably stable.

### Coarse-grained modeling

With the atomic structures of the pre- and post- power-stroke structures of dynein motor domain, we performed coarse-grained MD simulations of the entire motor domain to investigate conformational change pathways between the two structures. The coarse-grained model represents each amino acid as a single bead located at its Cα position. Dividing the entire motor domain into eight modules, we assumed that each module adopts two locally stable states corresponding to the pre- and post- power-stroke structures. Thus, we assigned a double-basin potential for each region/module. Formally, we can write the entire potential energy function as, *V*_*total*_ = ∑_*I* = 1,8_ *V*_*MB*,*I*_ where *V*_*MB*,*I*_ represents the multiple (two in this work) basin potential for the *I*-th modules partially including the interactions with their neighbors. (The explicit forms will be described below). Since each double basin potential possesses two basins, corresponding to the pre- and post- power-stroke states, the entire motor domain can have, in theory, 2^8^ local minima. Among all the minima, the one where all regions were in the pre- (or post-) power-stroke state coincides with the pre- (or post-) power-stroke structure of the entire motor domain. In this modeling, the entire conformational change occurs in a modular manner, region by region. From the pre-power-stroke to the post-power-stroke structure (and also in the recovery-stroke process), there can be 8! possible orders of transitions for every module.

### The atomic interaction-based coarse-grained (AICG2+) model

Before describing the double-basin model *V*_*MB*,*I*_, we start with the explanation of the energy function AICG2+ for a single basin model [47,48]. The AICG2+ potential is explicitly biased towards the reference structure, while all the terms are tuned to represent chemical interactions at the reference structure.

The potential energy function is written as

$$\begin{array}{l} V_{AICG2+}(R|R_{0})=\sum_{i}K_{b,i}{\left(b_{i}-b_{i,0}\right)}^{2}+V_{loc}^{flp}+\sum_{j=i+2}\varepsilon_{loc,ij}exp\left(-\frac{{\left(r_{ij}-r_{ij0}\right)}^{2}}{2W_{ij}^{2}}\right)\\ \;+\sum_{j=i+3}\varepsilon_{loc,ij}exp\left(-\frac{{\left(\phi_{ij}-\phi_{ij0}\right)}^{2}}{2W_{\phi ,ij}^{2}}\right)\\ \;+\sum_{i<j-3}^{nat\;contact}\varepsilon_{go,ij}\left[5{\left(\frac{r_{ij0}}{r_{ij}}\right)}^{12}-6{\left(\frac{r_{ij0}}{r_{ij}}\right)}^{10}\right]+\sum_{i<j-3}^{non-native}\varepsilon_{ev}{\left(\frac{d}{r_{ij}}\right)}^{12} \end{array}$$

Here, each term represents, in order, the elasticity of the virtual bond, the sequence-dependent angle- and dihedral-angle potential, the structure-based local potential between i-th and i+2-th residues, the structure-based local potential for dihedral angles, the Gō potential for non-local natively interacting pairs, and the generic repulsion for the rest of the non-local pairs. The vector ***R*** represents the 3*n*_*aa*_-dimensional Cartesian coordinates of the target protein where *n*_*aa*_ is the number of amino acids in the protein. ***R***_**0**_ is the corresponding coordinates in the reference structure (All the variables with the subscript 0 refer to parameters with their corresponding value from the reference structure). *b*_*i*_ is the i-th virtual bond length between i-th and i+1-th amino acids. $V_{loc}^{flp}$ is the sequence-dependent local potential [65]. *r*_*ij*_ is the distance between the i-th and j-th residues. *ϕ*_*ij*_ is the dihedral angle defined as i-th, i+1-th, i+2-th, and i+3-th residues. $W_{ij}^{2}$ and $W_{\phi ,ij}^{2}$ are the parameters representing the widths of the attractive interaction. The parameters *K*_*b*,*ibd*_, *ε*_*loc*,*ij*_, *ε*_*go*,*ij*_ are determined based on atomic-interactions evaluated by AMBER force field via a multiscale algorithm. The parameters *ε*_*ev*_ and *d* are determined from a structural survey. The CafeMol manual should be consulted for the meaning and the default values of these parameters (http://www.cafemol.org).

### Multiple-basin-model

Given the two single-basin potential functions *V*(***R***|***R***_***ν***_) with different reference structures ν = 1, 2, we define the multiple-basin potential for each module *V*_*MB*,*I*_ as the smaller eigenvalue of the eigenvalue equation,

$$\left(\begin{array}{cc} V_{I}(R|R_{1}) & \Delta \\ \Delta & V_{I}(R|R_{2})+\Delta V \end{array}\right)\left(\begin{array}{c} c_{1}\\ c_{2} \end{array}\right)=V_{MB,I}\left(\begin{array}{c} c_{1}\\ c_{2} \end{array}\right)$$

Here, the single basin potentials *V*_*I*_(***R***|***R***_***ν***_) for *I*-th modules are constructed from the AICG2+ model for each module with small modifications to adapt it with the multiple-basin model, as described in Okazaki et al [49]. Notably, we put interaction energies between neighboring modules in the multiple-basin potential of one module out of two interacting modules. The eigenvalue can be obtained by solving the secular equation.

We can explicitly write the smaller eigenvalue as

$$V_{MB,I}=\frac{V_{I}(R|R_{1})+V_{I}(R|R_{2})+\Delta V}{2}-\sqrt{{\left(\frac{V_{I}(R|R_{1})-V_{I}(R|R_{2})-\Delta V}{2}\right)}^{2}+\Delta^{2}}$$

Here, each multiple-basin potential contains two parameters, the coupling constant *Δ* that is related to the potential barrier height and *ΔV* that modulates the relative stability of the two basins (S1A Fig). For dynein, these parameters cannot be easily determined from experiments or in a bottom-up manner. Thus, we treat them as empirical parameters, the fine tuning of which is described in detail in the next subsection.

Using the eigenvector for the smaller eigenvalue, we can define the reaction coordinate as *χ* = ln(*c*_2_/*c*_1_) which takes the negative (positive) value when the system stays in the basin 1 (2).

As mentioned above, we divided the dynein motor domain into eight modules. Thus, the entire motor domain is described by eight multiple-basin potentials. For each module, we included the intra-module interaction and some inter-module interactions associated with the module into the multiple-basin potential (see S1 Table). Each inter-module interaction term is included in only one of the relevant multiple-basin potential systems so that no double counting occurs. Notably, even though the inter-module interaction is included only in one of two multiple-basin potentials, the coarse-grained MD is conducted by using the summation of all the terms so that the inter-module interaction affects conformational changes in both modules. Transitions in the regions/modules are described by *χ* values. Based on the sign of the *χ*, we can assign if the module is either in the pre-power-stroke state or in the post-power-stroke state. By combination, the entire motor domain can have, in theory, 2^8^ states.

### Parameter determination

Although the values of parameters *Δ* and *ΔV* can, in principle, be determined from all atom molecular mechanics calculations or from experimental data, if available, we cannot currently use these approaches for the dynein motor domain. In this study, we tuned these parameters purely empirically via many preliminary simulations, as described below.

The parameter *Δ* modulates the energy barrier height between two basins. When one uses a small value of *Δ*, there is no observable conformational transition over a feasible computer time frame (An example is shown in S1B Fig top right panel). Conversely, at an extremely large value of the parameter *Δ*, the two basins are completely coalesced to form a single basin between the positions of the two reference structures (S1B Fig top left). Neither of these situations is desirable. Thus, we sought a range for the value of the *Δ* parameter, in which we could observe conformational transitions within the possible simulation time. In general, the range of acceptable *Δ* values is relatively narrow and thus we can determine the value of *Δ* with little uncertainty. The *Δ* parameter values thus decided are listed in S6 Table.

The parameter *ΔV* modulates the relative stability of the two basins. Here, we defined *ΔV* as the energy shift in the final structure, relative to the initial structure. For example, in the power-stroke process, the final structure is the post-power-stroke state and thus an increase in the value of *ΔV* destabilizes the post-power-stroke state relative to the pre-power-stroke state. When one uses a very small *ΔV* value (often a negative value), one normally observes the conformational transition immediately at the beginning of simulations (as exemplified in S1B Fig bottom left). When one adopts a very large *ΔV* value, the transition is never observed (S1B Fig bottom right). Neither of these situations is desirable. For each of the power-stroke and recovery-stroke processes, we tuned the value of *ΔV* so that we could observe the conformational transition in the middle of the simulation time window. As was the case for *Δ*, the range of acceptable *ΔV* values was relatively narrow and so we could also assign values with confidence.

We found one set of these values that match all these criteria, and refer to it as the standard set. Since the choice of parameters is far from unique, we comprehensively investigated the sensitivity/robustness of the results when we alter the parameter values *ΔV*. Specifically, relative to the standard set, we introduced either a destabilization in the final state (retarding effect), or a stabilization in the final state (accelerating effect) into each region/module, by the *ΔV* change of ±10 kcal/mol (S2 Table for the power-stroke and S3 Table for the recovery-stroke processes).

Conformational change simulations were performed 30 times with different stochastic forces for the standard parameter set and 10 times for the other sets that introduced a retarding or accelerating effect.

### Coarse-grained MD simulations

We utilized the underdamped Langevin dynamics for constant temperature MD simulations. The friction coefficient was 0.02 (in CafeMol units) and the temperature was set at 300 K. Each MD simulation consists of 10^7^ MD steps. One MD step was mapped very roughly to ~1 ps [45]. Thus, each trajectory corresponds to approximately 10 μs although the time mapping was a little inaccurate, especially because the transition rates depend on the empirical parameter *Δ*. All of the coarse-grained MD simulations were performed using an in-house modified version of CafeMol 2.1.

### How to reconstruct all-atom structural model

To infer atomic interaction at the interface between modules, we modeled the all-atom structures of representative intermediate states. The reconstruction method is the same as that used to model the pre/post structural models. First, PD2 ca2main [59] were used to reconstruct the atomic model for the backbone and we also did energy minimization as an option. Then, Scwrl4 [60] were used to reconstruct the atomic model for the sidechain. Additionally, we checked the stability of these reconstructed structures by performing short all-atom MD simulations from these structures. The setup of simulation is the same as that for the pre/post structures’ case (S11 Fig).

## Supporting information

S1 Fig Multiple basin model. (A) A cartoon of the multiple (double) basin model. The horizontal and vertical axes represent conformational space and the effective energy, respectively. The dashed curves are the single-basin potential, from the double basin model (the solid curve) is defined via two parameters. The parameter *Δ* lowers the energy barrier between the two basins. The parameter Δ*V* modulates the relative energy of basin 2 relative to that of basin 1. (B) Examples of results obtained by assigning inappropriate values to the parameters. (Top left) the parameter Δ for the AAA4 double basin model set as too large a value. (Top right) the parameter Δ for the AAA4 double basin model set as too small a value. (Bottom left) the parameter Δ*V* set as too large (negative) a value. (Bottom right) Δ*V* is set as too small a value. (C) An example of trajectories when the interaction between the linker and the AAA1 module was included into the AAA1 system. This interaction was included in the linker system in the default setups.
(EPS)

S2 Fig Statistical significance in order-map. We examined statistical significance of P-order deduced from 30 trajectories in the standard setup. We calculated the *p*-values for the null hypothesis that for each pair of the transitions, P-order appears by chance. The left and right panels are for the power stroke and the recovery stroke, respectively. The lower panels represent p-values, while the upper panels are the same as those given in Fig 2B and Fig 4B. We can confirm that, in the power-stroke, most *p*-values are rather small except the column/row of AAA2 which are large so that these orders are insignificant. In the recovery-stroke, because the clockwise and the anti-clockwise propagations proceed simultaneously, *p*-values between the clockwise and anti-clockwise pathways tend to be large.
(EPS)

S3 Fig The bootstrap analysis of pair rate constants. In this work, we used pair rate constants to monitor the correlation between two events (transitions). The average value of the pair rate constants were calculated from 30 (the standard setups) or 10 (the other setups) trajectories. Here we estimate the uncertainty (statistical error) of the average values, using a bootstrap analysis. From 30 trajectories of the standard setups, we randomly drew 30 samples, allowing the multiplications, and calculated the average. Repeating it 2000 times, we obtained the histogram of the average, from which we got the 95% confidence interval. The 95% confidence interval for the pair rate of the linker→AAA1 transitions in the power stroke is [0.065, 0.13] (A), that for the AAA3→AAA4 transitions in the power stroke is [0.0060, 0.011] (B), that for the AAA2→linker transitions in the recovery stroke is [0.0041, 0.023] (C), and that for the AAA2→AAA3 transition in the recovery stroke is [0.0044, 0.018] (D).
(EPS)

S4 Fig Comparison of the time-difference distribution between the perfectly-independent models and MD simulation data. Under the assumption that the two events A and B are perfectly independent, we obtained the analytical distribution of the time difference Δ*t* between the two transitions (the red curve). (See S1 Text for the analytical form). The histogram in black is the Δ*t* result from the 30 MD trajectories. (A) The linker and AAA1 transitions in the power-stroke pathway. (B) The AAA3 and AAA4 transitions in the power-stroke pathway (C) The linker and AAA2 transitions in the recovery stroke pathway (D) The AAA2 and AAA3 transitions in the recovery stroke pathway. The “null” hypothesis that the histogram is drawn from the theoretical distribution can be tested by the one-sample Kolmogorov-Smirnov test (KS test), which is presented in S4 Table.
(EPS)

S5 Fig Order maps of all the power-stroke simulations. The description of each figure is the same as in Fig 2B. The integer number labels correspond to the setup numbers in S2 Table.
(EPS)

S6 Fig Multiple sequence alignment. To investigate the sequence conservation in the inter-module interface within dynein-1 family and between dynein-1 and -2, we performed a multiple sequence alignment. First, the homology search was performed using BLAST with the *Dictyostelium discoideum* dynein-1 sequence as a query. Subsequently, clustering was performed using CD-Hit on a total of 31 sequences of the top 30 sequences close to the query plus one human dynein-2 sequence. Multiple sequence alignment was performed by clustal-omega using the query sequence, 10 more sequences of dynein-1, and the one from human dynein-2. The sequence identity between the *Dictyostelium discoideum* dynein-1 (sequence 1) and the human dynein-2 (sequence 12) was 28%. The alignment results around the residues of interest in the text are shown in this figure.
The portion of sequence in (A) corresponds to Fig 3B. The site 1861 is highly conserved allowing only D and E between dynein-1 and -2. E1849 in the linker acting on Q2116 of AAA1 during the pre structure was well conserved in dynein-1 suggesting its importance although it is mutated in human dynein-2. The portion of sequence in (B) corresponds to Fig 5B. It was confirmed that all of the residues of interest were completely conserved across the families. The portion of sequence in (C) corresponds to Fig 5C. While these residues are well conserved in dynein-1 suggesting its importance in dynein-1 family, this residue was not preserved in dynein-2. The portion of sequence in (D) corresponds to S10 Fig. Within dynein-1 family, these sites are well conserved, while unfortunately, this part also has low conservation in dynein-2.
(EPS)

S7 Fig Pair rate maps of all the power-stroke simulations. The description of each figure is the same as in Fig 2C. The integer number labels correspond to the setup numbers in S2 Table.
(EPS)

S8 Fig Order maps of all the recovery-stroke simulations. The description of each figure is the same as in Fig 4B. The integer number labels correspond to the setup numbers in S3 Table.
(EPS)

S9 Fig Pair rate maps of all the recovery-stroke simulations. The description of each figure is the same as in Fig 4C. The integer number labels correspond to the setup numbers in S3 Table.
(EPS)

S10 Fig A close up view of the interface between the linker (purple) and the AAA3 module (light green) in the pre-power-stroke structure (left) and a snapshot at 220 x 104 MD step. From the pre-power-stroke structure, the AAA3 module changed its structure so that a loop in AAA3 (T2723, P2724) moved away from the interface residues to the linker (L1569, D1592, N1596). Representative distances are shown in Ǻ.
(EPS)

S11 Fig Stability of reference and intermediate structures investigated by all-atom MD. From all-atom models of the pre- and post- power stroke structures and four intermediate structures, we performed all-atom MD to address their short-time stability. The root-mean-square-deviations (RMSDs) from the initial structures are plotted as a function of MD time for 10 ns. The RMSD was calculated only for the inker plus the AAA+ ring. In all the cases, the RMSD saturated near 0.5 nm. Given the large size of the molecule, we consider these structures reasonably stable locally.
(EPS)

S1 Movie Power stroke pathway. (MP4)

S2 Movie Recovery stroke pathway. (MP4)

S1 Table Eight systems of multiple-basin-models. (PDF)

S2 Table Multiple-basin model parameters Δ*V* in the forward powerstroke simulations. ↓ and ↑ mean retarding and accelerating effects, respectively to the indicated regions.
(PDF)

S3 Table Multiple-basin model parameters Δ*V* in the recovery stroke simulations. ↓ and ↑ mean retarding and accelerating effects, respectively to the indicated regions.
(PDF)

S4 Table One-sample Kolmogorov–Smirnov test for the probability distribution of the time-difference between two transition events. The time-difference Δ*t* data between the two transitions in MD trajectories were compared with the analytical time-difference distribution for the hypothetical perfectly-independent model (“null” hypothesis) by the Kolmogorov-Smirnov test (More explanation in S1 Text). The top left (bottom right) triangles are for the power-stroke (recovery stroke) pathways. The numbers given are the maximum deviation *D* in the cumulative density functions/histograms. With the data size 30, the independence (“null”) hypothesis can be denied with the 95% confidence if *D* is larger than 0.2417 (marked red).
(PDF)

S5 Table Residue contact numbers between 8 regions in pre- and post powerstroke structures. The top left (bottom right) triangles are for the pre-powerstroke (post-powerstroke) structures. The residue contact between *i* and *j* is defined to be made if at least one atom in the residue *i* is within 6.5Ǻ to one atom in the residue *j*.
(PDF)

S6 Table Multiple-basin-model parameters Δ. (PDF)

S1 Text The treatment of the rate-constant. (PDF)
