# Supplementary material for: Allosteric conformational change cascade in cytoplasmic dynein revealed by structure-based molecular simulations
Source: PLoS Comput Biol. 2017 Sep 11;13(9):e1005748. doi: 10.1371/journal.pcbi.1005748 (PMC5608440; doi:10.1371/journal.pcbi.1005748)
Supplement: S1 Table — (PDF) [file pcbi.1005748.s014.pdf]

S1 Table. Eight systems of multiple-basin-models

	Interaction energy included	
system ID	Intra-domain	Inter-domain
1	linker	linker/(AAA1, AAA2, AAA3, AAA4, AAA5, AAA6-C-terminal)
2	AAA1	AAA1/(AAA2, AAA3)
3	AAA2	AAA2/AAA3
4	AAA3	AAA3/AAA4
5	AAA4	AAA4/AAA5
6	MTBD	MTDB/(AAA4, AAA5)
7	AAA5	AAA5/AAA6-C-terminal
8	AAA6-C-terminal	(AAA1, AAA2, AAA3, AAA4)/AAA6-C-terminal
