# Supplementary material for: Allosteric conformational change cascade in cytoplasmic dynein revealed by structure-based molecular simulations
Source: PLoS Comput Biol. 2017 Sep 11;13(9):e1005748. doi: 10.1371/journal.pcbi.1005748 (PMC5608440; doi:10.1371/journal.pcbi.1005748)
Supplement: S2 Table — ↓ and ↑ mean retarding and accelerating effects, respectively to the indicated regions. (PDF) [file pcbi.1005748.s015.pdf]

S2 Table. Multiple-basin model parameters Δ*V* in the forward powerstroke simulations

(ΔV, kcal/mol)

Setup	system1 linker	system2 AAA1	system3 AAA2	system4 AAA3	system5 AAA4	system6 MTBD	system7 AAA5	system8 AAA6
1(standard)	-20	-5	-120	-5	-30	-65	-90	-200
2(linker ↓)	**-10**	-5	-120	-5	-30	-65	-90	-200
3(linker ↑)	**-30**	-5	-120	-5	-30	-65	-90	-200
4(AAA1 ↓)	-20	**5**	-120	-5	-30	-65	-90	-200
5(AAA1 ↑)	-20	**-15**	-120	-5	-30	-65	-90	-200
6(AAA3 ↓)	-20	-5	-120	**5**	-30	-65	-90	-200
7(AAA3 ↑)	-20	-5	-120	**-15**	-30	-65	-90	-200
8(AAA4 ↓)	-20	-5	-120	-5	**-20**	-65	-90	-200
9(AAA4 ↑)	-20	-5	-120	-5	**-40**	-65	-90	-200
10(AAA1 ↓↓)	-20	**50**	-120	-5	-30	-65	-90	-200
11(AAA2 ↓)	-20	-5	**-110**	-5	-30	-65	-90	-200
12(AAA2 ↑)	-20	-5	**-130**	-5	-30	-65	-90	-200
13(AAA5 ↓)	-20	-5	-120	-5	-30	-65	**-80**	-200
14(*AAA*5 ↑)	-20	-5	-120	-5	-30	-65	**-100**	-200
15(AAA6 ↓)	-20	-5	-120	-5	-30	-65	-90	**-190**
16(AAA6 ↑)	-20	-5	-120	-5	-30	-65	-90	**-210**
17(MTBD ↓)	-20	-5	-120	-5	-30	**-55**	-90	-200
18(MTBD ↑)	-20	-5	-120	-5	-30	**-75**	-90	-200

↓ and ↑ mean retarding and accelerating effects, respectively to the indicated regions.
