# Supplementary material for: Allosteric conformational change cascade in cytoplasmic dynein revealed by structure-based molecular simulations
Source: PLoS Comput Biol. 2017 Sep 11;13(9):e1005748. doi: 10.1371/journal.pcbi.1005748 (PMC5608440; doi:10.1371/journal.pcbi.1005748)
Supplement: S3 Table — ↓ and ↑ mean retarding and accelerating effects, respectively to the indicated regions. (PDF) [file pcbi.1005748.s016.pdf]

S3 Table. Multiple-basin model parameters Δ*V* in the recovery stroke simulations

(ΔV, kcal/mol)

setup	system1 linker	system2 AAA1	system3 AAA2	system4 AAA3	system5 AAA4	system6 MTBD	system7 AAA5	system8 AAA6
1(standard)	-200	-57	-380	-90	-100	-60	-325	-295
2(AAA6 ↓)	-200	-57	-380	-90	-100	-60	-325	**-285**
3(AAA6 ↑)	-200	-57	-380	-90	-100	-60	-325	**-305**
4(AAA5 ↓)	-200	-57	-380	-90	-100	-60	**-315**	-295
5(AAA5 ↑)	-200	-57	-380	-90	-100	-60	**-335**	-295
6(MTBD ↓)	-200	-57	-380	-90	-100	**-50**	-325	-295
7(MTBD ↑)	-200	-57	-380	-90	-100	**-70**	-325	-295
8(AAA4 ↓)	-200	-57	-380	-90	**-90**	-60	-325	-295
9(AAA4 ↑)	-200	-57	-380	-90	**-110**	-60	-325	-295
10(AAA3 ↓)	-200	-57	-380	**-80**	-100	-60	-325	-295
11(AAA3 ↑)	-200	-57	-380	**-100**	-100	-60	-325	-295
12(AAA2 ↓)	-200	-57	**-370**	-90	-100	-60	-325	-295
13(AAA2 ↑)	-200	-57	**-390**	-90	-100	-60	-325	-295
14(AAA1 ↓)	-200	**-47**	-380	-90	-100	-60	-325	-295
15(AAA1 ↑)	-200	**-67**	-380	-90	-100	-60	-325	-295
16(linker ↓)	**-190**	-57	-380	-90	-100	-60	-325	-295
17(linker ↑)	**-210**	-57	-380	-90	-100	-60	-325	-295
