# Supplementary material for: Allosteric conformational change cascade in cytoplasmic dynein revealed by structure-based molecular simulations
Source: PLoS Comput Biol. 2017 Sep 11;13(9):e1005748. doi: 10.1371/journal.pcbi.1005748 (PMC5608440; doi:10.1371/journal.pcbi.1005748)
Supplement: S4 Table — The time-difference Δt data between the two transitions in MD trajectories were compared with the analytical time-difference distribution for the hypothetical perfectly-independent model (“null” hypothesis) by the Kolmogorov-Smirnov test (More explanation in S1 Text). The top left (bottom right) triangles are for the power-stroke (recovery stroke) pathways. The numbers given are the maximum deviation D in the cumulative density functions/histograms. With the data size 30, the independence (“null”) hypothesis can be denied with the 95% confidence if D is larger than 0.2417 (marked red). (PDF) [file pcbi.1005748.s017.pdf]

S4 Table. One-sample Kolmogorov - Smirnov test for the probability distribution of the time-difference between two transition events

AAA6	0.39	0.40	0.36	0.41	0.40	0.41	0.47	
AAA5	0.34	0.36	0.17	0.35	0.41	0.40		0.37
MTBD	0.23	0.24	0.17	0.30	0.39		0.40	0.34
AAA4	0.22	0.25	0.20	0.31		0.38	0.36	0.39
AAA3	0.18	0.21	0.26		0.35	0.27	0.27	0.35
AAA2	0.30	0.33		0.40	0.36	0.18	0.17	0.20
AAA1	0.45		0.21	0.36	0.39	0.36	0.37	0.43
Linker		0.33	0.29	0.16	0.19	0.27	0.22	0.33
	Linker	AAA1	AAA2	AAA3	AAA4	MTBD	AAA5	AAA6
