# Supplementary material for: Allosteric conformational change cascade in cytoplasmic dynein revealed by structure-based molecular simulations
Source: PLoS Comput Biol. 2017 Sep 11;13(9):e1005748. doi: 10.1371/journal.pcbi.1005748 (PMC5608440; doi:10.1371/journal.pcbi.1005748)
Supplement: S5 Table — The top left (bottom right) triangles are for the pre-powerstroke (post-powerstroke) structures. The residue contact between i and j is defined to be made if at least one atom in the residue i is within 6.5Ǻ to one atom in the residue j. (PDF) [file pcbi.1005748.s018.pdf]

S5 Table. Residue contact numbers between 8 regions in pre- and post powerstroke structures

AAA6	34	253	44	35	29	71	258	
AAA5	0	0	0	0	209	61		272
MTBD	0	0	0	0	123		108	0
AAA4	0	0	0	216		141	189	43
AAA3	37	4	376		192	0	0	13
AAA2	58	230		315	0	0	0	0
AAA1	290		128	0	0	0	0	207
Linker		317	29	0	4	0	5	23
	Linker	AAA1	AAA2	AAA3	AAA4	MTBD	AAA5	AAA6
The number of amino acids	414	294	402	317	356	334	476	616
