# Supplementary material for: Allosteric conformational change cascade in cytoplasmic dynein revealed by structure-based molecular simulations
Source: PLoS Comput Biol. 2017 Sep 11;13(9):e1005748. doi: 10.1371/journal.pcbi.1005748 (PMC5608440; doi:10.1371/journal.pcbi.1005748)
Supplement: S6 Table — (PDF) [file pcbi.1005748.s019.pdf]

S6 Table. Multiple-basin-model parameters Δ

(Δ, kcal/mol)

	System1 linker	system2 AAA1	system3 AAA2	system4 AAA3	system5 AAA4	system6 MTBD	system7 AAA5	system8 AAA6
Δ	985	650	1200	680	800	885	980	1550
