# Supplementary material for: Allosteric conformational change cascade in cytoplasmic dynein revealed by structure-based molecular simulations
Source: PLoS Comput Biol. 2017 Sep 11;13(9):e1005748. doi: 10.1371/journal.pcbi.1005748 (PMC5608440; doi:10.1371/journal.pcbi.1005748)
Supplement: S1 Text — (PDF) [file pcbi.1005748.s020.pdf]

S1 Text. The pair rate constant

The pair rate constant is simple to define but it is difficult to interpret. Here, we present some analysis.

First, we repeat the definition. The average pair rate constants $k(\mathrm{b|a})$, for the transition in "b" after the transition of "a", is defined as

$k(\mathrm{b|a}) \quad = (1/n) \sum_{i=1}^{n} 1/\Delta t_{ab}(i)$

where the summation is taken over the trajectories in which "b" transited later than "a". $\Delta t_{ab}(i)$ is the time interval from the transition in a to that in b in the i-th trajectory, and n is the number of the corresponding trajectories. When transition "b" did not occur, we set the time of the "b" transition as the final time of the simulation. The rate is given in the unit $1/10^4$ MD steps

We used the pair rate to infer the dependence of the two events. Namely, if the pair rate is large, we interpret that the transition in b tends to be dependent on a. Of course, this is not straightforward because the intrinsic transition rate of b may simply be just slightly smaller than that of a so that the transition in b tends to occur slightly after that of a, which is nothing to do with the "dependence". One way to detect the dependence is to give a perturbation to the transition in a, either positively (i.e., acceleration) or negatively (deceleration). If that perturbation affects the transition in b, we can safely suggest the dependence. This is the logic mostly used in the main text.

Here, we provide one more rigorous analysis. We start with a hypothetical perfectly-independent two probabilistic transitions A and B that exhibit single-exponential kinetic, of which the transition rates coincide with those of A and B in real MD simulations. The time difference Δ between the transitions A and B takes the analytical probability distribution,

$$P(\Delta) = \int_0^{\infty} dt_1 \int_0^{\infty} dt_2 \, \delta[(t_1 - t_2) - \Delta] \, e^{-k_a t_1} e^{-k_b t_2}$$

We can solve it to obtain

$$P(\Delta) = \frac{k_a k_b}{k_a + k_b} e^{k_b \Delta}$$

for $\Delta < 0$, and

$$P(\Delta) = \frac{k_a k_b}{k_a + k_b} e^{-k_a \Delta}$$

for $\Delta \geq 0$.

We can compare this independent model with the real data of the time difference from the 30 trajectories, which is given in S4. Fig. Moreover, these distribution and histogram can be statistically tested with the one-sample Kolmogorov-Smirnov test (S4.Table). The numbers given in the table are the maximum deviation D in the cumulative density functions/histograms. With the data size 30, the independence ("null") hypothesis can be denied with the 95% confidence if D is larger than 0.2417. A number of pairs of transitions are recognized as "dependent" (marked red). Moreover, these data are consistent with the inference given above based on the average value of the pair rate constants and the analysis of the perturbed system.
